# Teaching Neuro*Images*: Neuroradiologic evolution of Leigh disease

**DOI:** 10.1212/WNL.0000000000003182

**Published:** 2016-10-04

**Authors:** Yi Shiau Ng, Ming Lim, Gareth Thomas, Robert McFarland

**Affiliations:** From the Wellcome Trust Centre for Mitochondrial Research (Y.S.N., R.M.), Institute of Neuroscience, Newcastle University, Newcastle upon Tyne; Evelina London Children's Hospital and Children's Neurosciences Centre, Newcomen Centre at St Thomas' Hospital (M.L.), London; and Morriston Hospital (G.T.), Abertawe Bro Morgannwg University Health Board, Wales, UK.

A 2-year-old girl with no significant family history presented with motor developmental delay and strabismus. MRI revealed unilateral basal ganglia and brainstem lesions (figure 1). Eighteen months later, she developed acute onset right arm weakness, leading to a diagnosis of multiphasic disseminated encephalomyelitis. Treatment with steroids and mycophenolate produced no symptomatic or imaging improvement. Her condition progressed, with ataxia, multifocal dystonia, spasticity, and loss of ambulation at age 6 years. Serial imaging showed evolution of bilateral basal ganglia changes, compatible with Leigh syndrome^1^ (figure 2). A heteroplasmic mutation, m.12706T>C in the *MTND5* gene, was identified in muscle.

**Figure 1:**
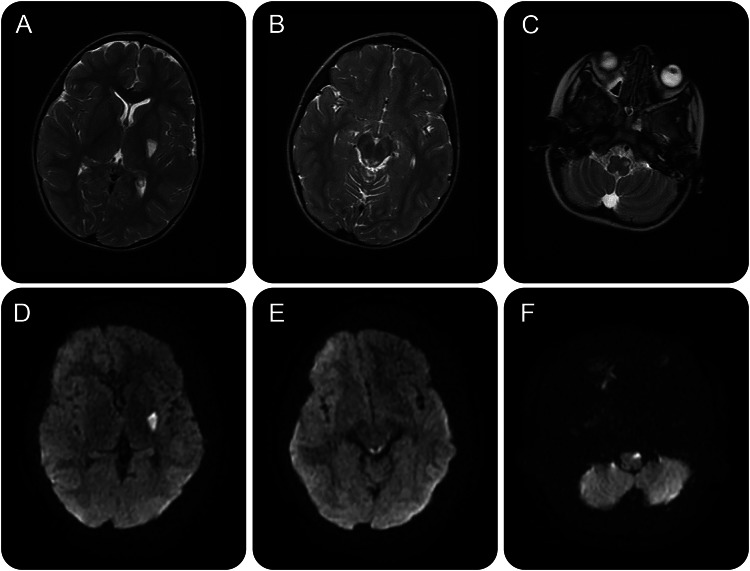
MRI head (age 2 years) Axial T2-weighted images (A–C) reveal hyperintensities involving the left globus pallidus, midbrain, and medulla, with diffusion restriction evident on diffusion-weighted sequences (D–F).

**Figure 2:**
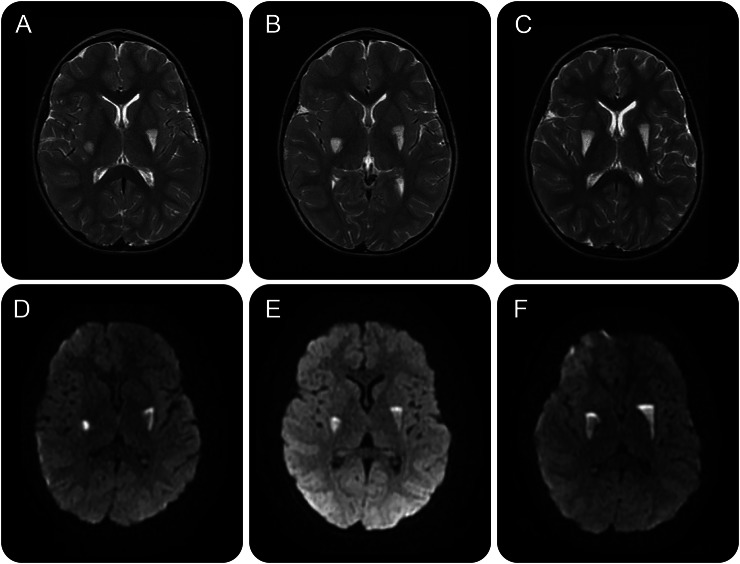
Serial MRI head images (T2 and diffusion-weighted) Serial T2 (A–C) and diffusion-weighted (D–F) images reveal an evolving hyperintense lesion in the right globus pallidus (age 4 years). This becomes symmetrical by age 5–6 years.

## Supplementary Material

Teaching Slides
