# Supplementary material for: Teaching NeuroImages: Neuroradiologic evolution of Leigh disease
Source: Neurology. 2016 Oct 4;87(14):e159–60. doi: 10.1212/WNL.0000000000003182 (PMC5075973; doi:10.1212/WNL.0000000000003182)
Supplement: Teaching Slides [file supp_87_14_e159__index.html]

Teaching Slides 

# Teaching Neuro*Images*: Neuroradiologic evolution of Leigh disease

## Teaching Slides

**Neurology® data supplements are not copyedited before publication. Published editorials and translations have been copyedited.  
 © 2016 American Academy of Neurology.  
  
 Files in this Data Supplement:**

- Teaching Slides - PowerPoint Presentation
