# Supplementary material for: Teaching NeuroImages: Neuroradiologic evolution of Leigh disease
Source: Neurology. 2016 Oct 4;87(14):e159–60. doi: 10.1212/WNL.0000000000003182 (PMC5075973; doi:10.1212/WNL.0000000000003182)
Supplement: Teaching Slides [file supp_WNL.0000000000003182_Teaching_slides.pptx]

## Slide 1
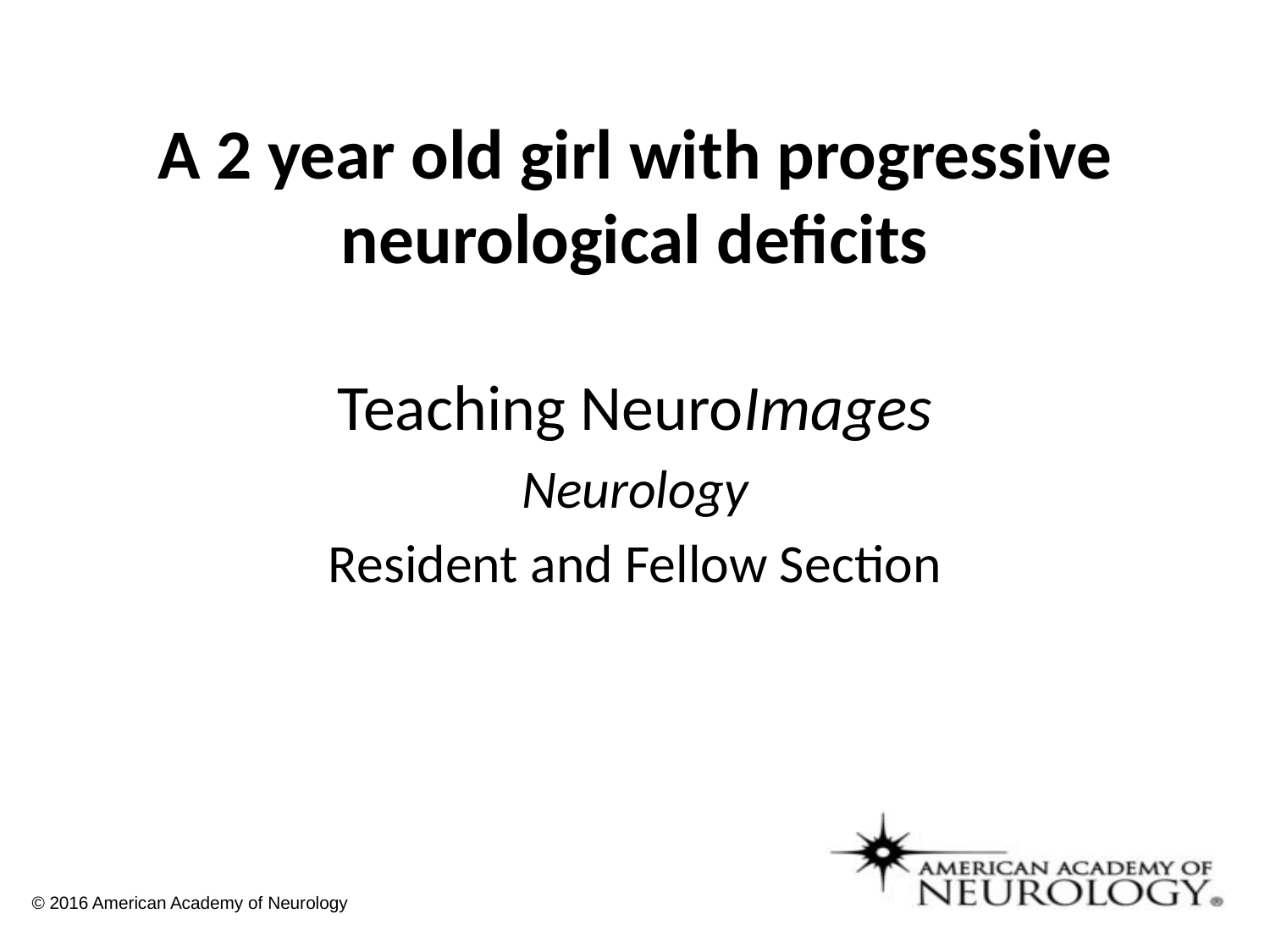

# A 2 year old girl with progressive neurological deficits
Teaching NeuroImages
Neurology
Resident and Fellow Section
© 2016 American Academy of Neurology

## Slide 2
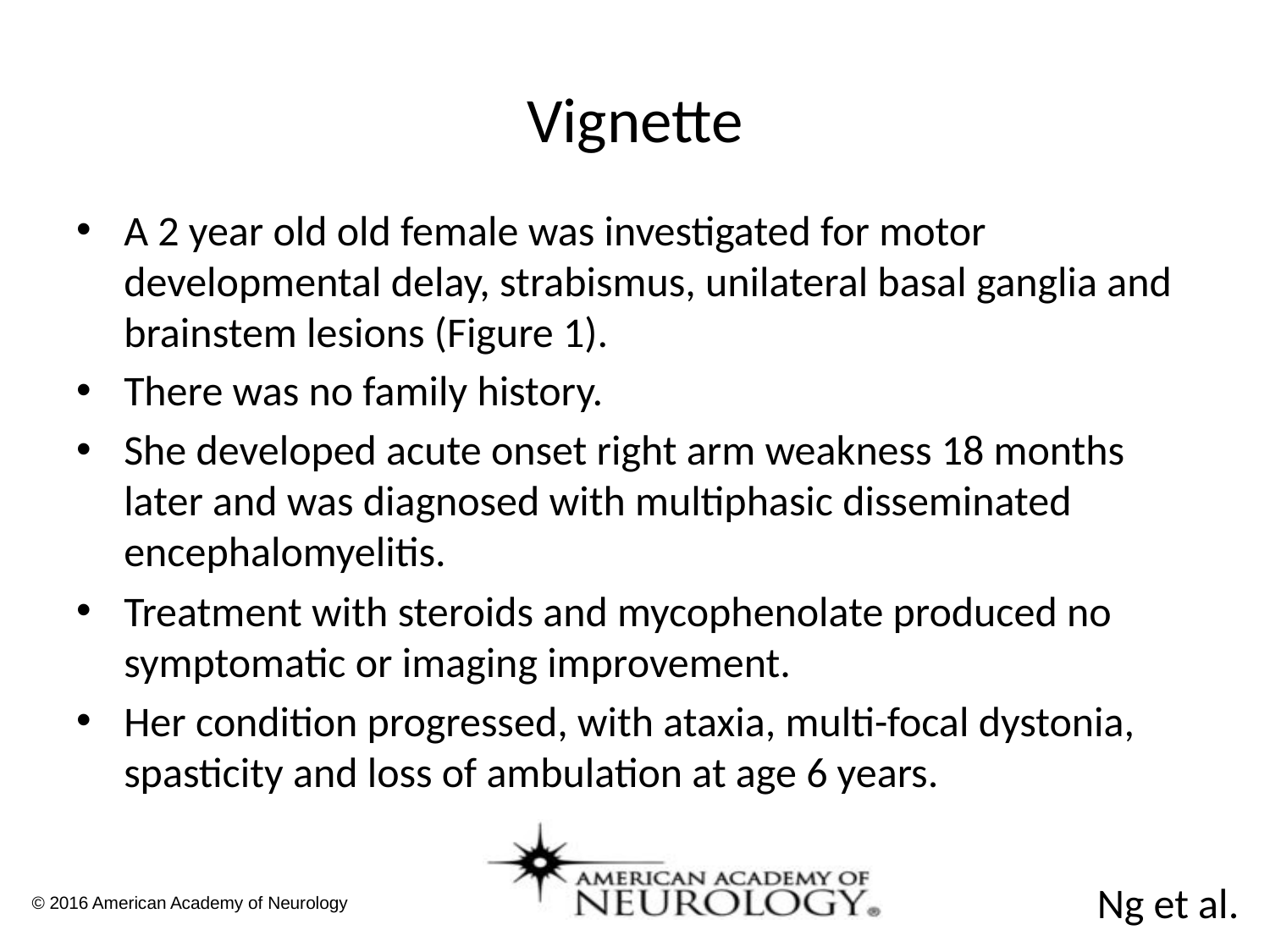

# Vignette
A 2 year old old female was investigated for motor developmental delay, strabismus, unilateral basal ganglia and brainstem lesions (Figure 1).
There was no family history.
She developed acute onset right arm weakness 18 months later and was diagnosed with multiphasic disseminated encephalomyelitis.
Treatment with steroids and mycophenolate produced no symptomatic or imaging improvement.
Her condition progressed, with ataxia, multi-focal dystonia, spasticity and loss of ambulation at age 6 years.
Ng et al.
© 2016 American Academy of Neurology

## Slide 3
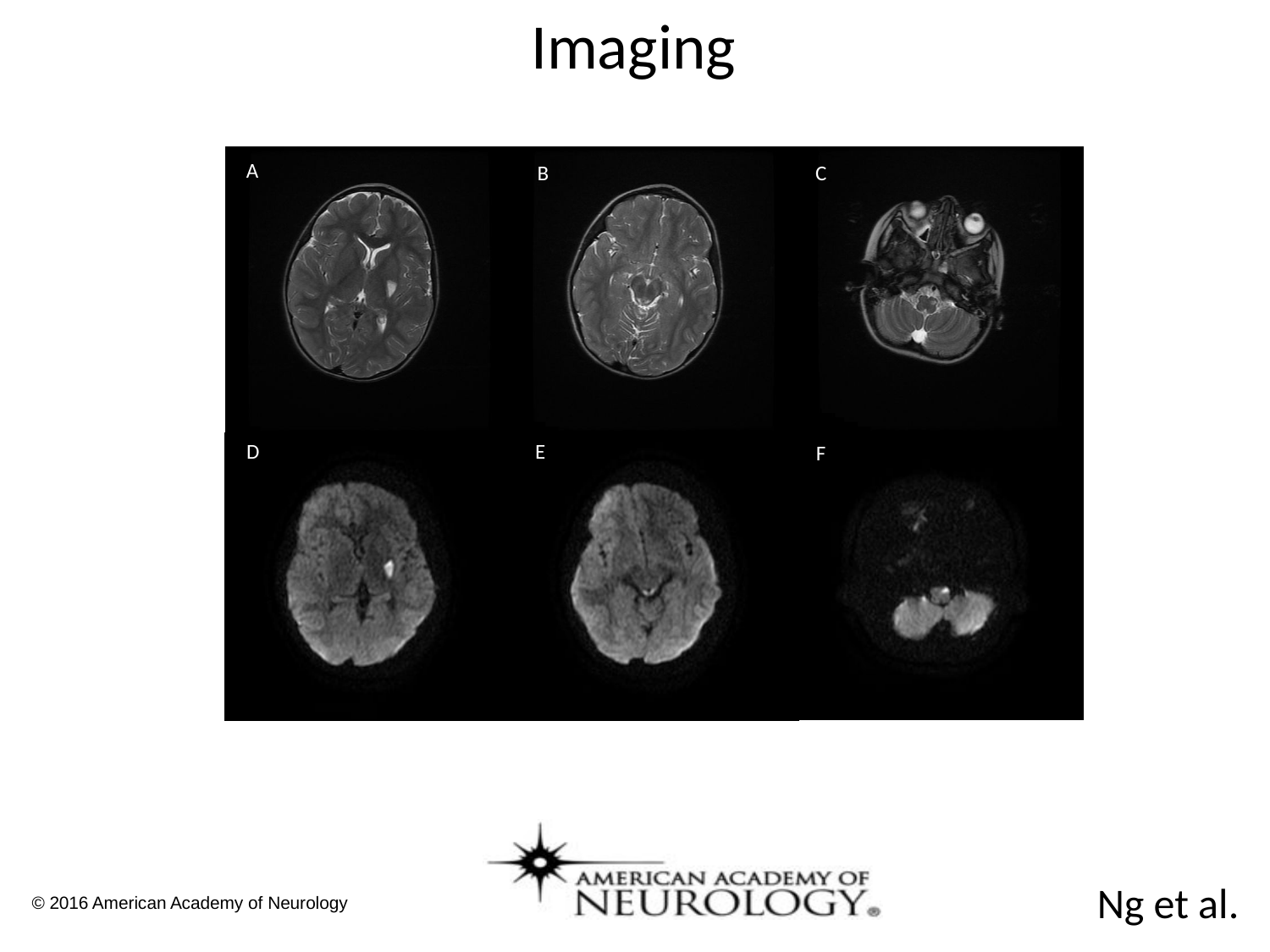

# Imaging
A
C
B
D
E
F
Ng et al.
© 2016 American Academy of Neurology

## Slide 4
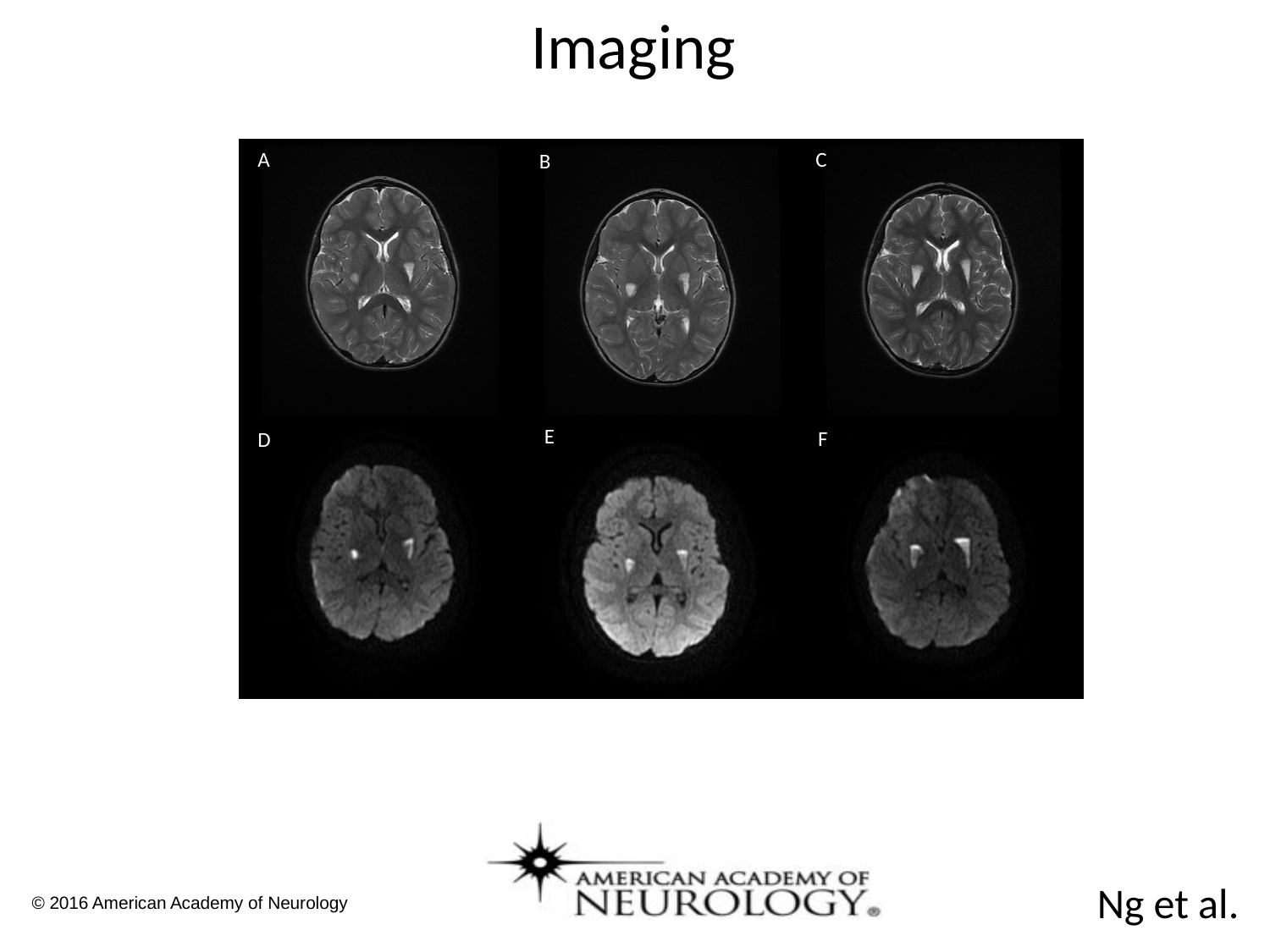

# Imaging
C
A
B
D
E
F
Ng et al.
© 2016 American Academy of Neurology

## Slide 5
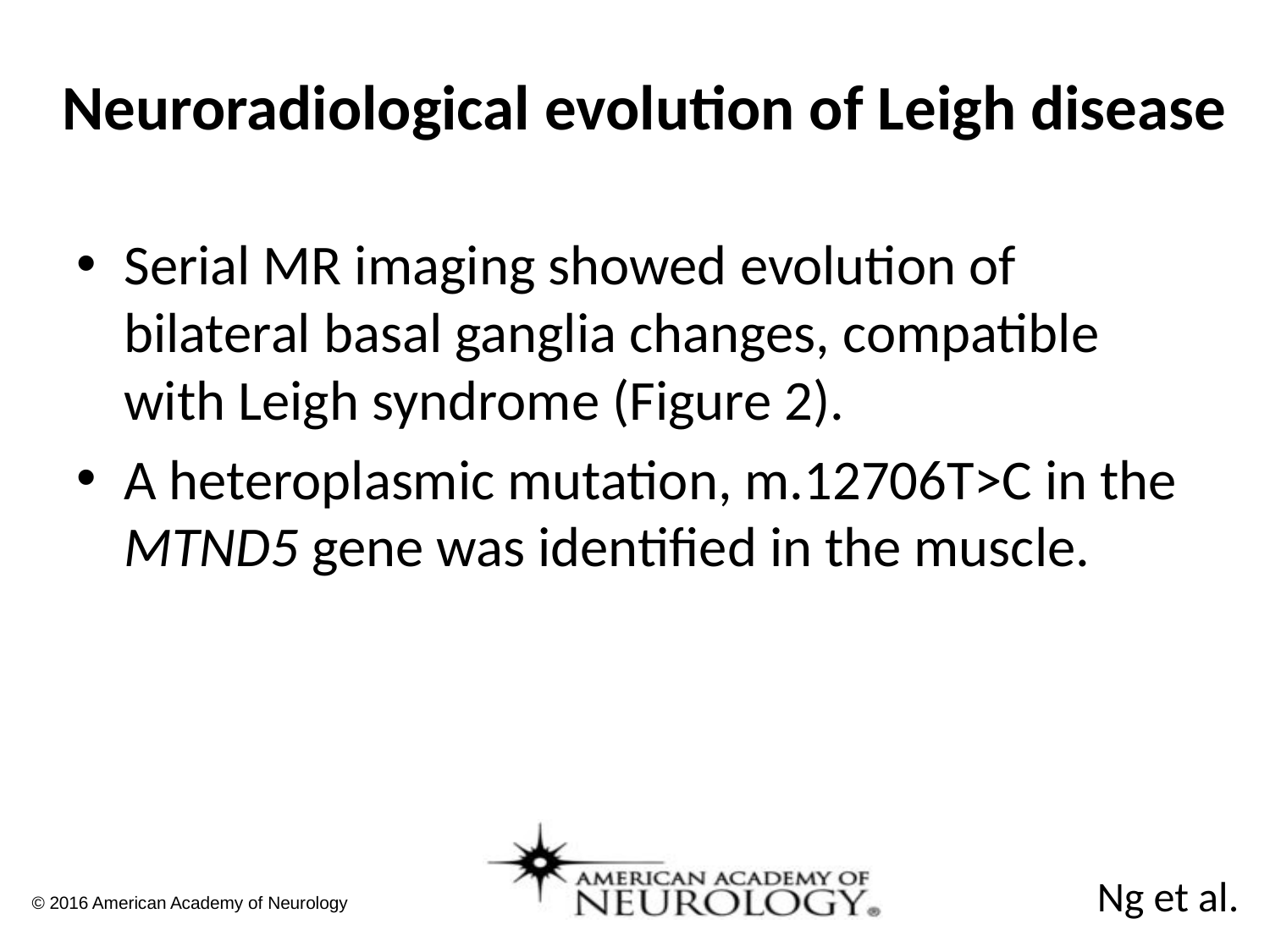

# Neuroradiological evolution of Leigh disease
Serial MR imaging showed evolution of bilateral basal ganglia changes, compatible with Leigh syndrome (Figure 2).
A heteroplasmic mutation, m.12706T>C in the MTND5 gene was identified in the muscle.
Ng et al.
© 2016 American Academy of Neurology
